# EXPLORING DEPRESSION AND ITS CORRELATES AMONG RESETTLED BHUTANESE OLDER ADULTS IN OHIO

**DOI:** 10.1093/geroni/igac059.2872

**Published:** 2022-12-20

**Authors:** Saruna Ghimire, Bunsi Chapadia, Isha Karmacharya, Aman Shrestha, Janardan Subedi, Robert Applebaum

**Affiliations:** Miami University, Oxford, Ohio, United States; Miami University, Oxford, Ohio, United States; Miami University, Oxford, Ohio, United States; Miami University, Oxford, Ohio, United States; Miami University, Oxford, Ohio, United States; Miami University, Oxford, Ohio, United States

## Abstract

Following the “ethnic cleansing” by the Bhutanese government in the 1990s, Nepali-lingual Bhutanese fled from southern Bhutan and spent about two decades in refugee camps in Nepal before resettlement in the US and other countries. During post-resettlement, this population had a high rate of suicide and mental health problems. However, studies specifically among resettled older Bhutanese are lacking. This study aims to estimate the prevalence of depression and explore its correlates among resettled Bhutanese Older Adults in Ohio. A cross-sectional survey was conducted in Ohio (Columbus, Akron, Cleveland, and Cincinnati). The exploratory study surveyed 275 participants between January-June 2022, using snowball sampling, given the absence of a sampling frame. Study participants include adults 55 years and above, identified with the help of local community-based organizations. Depression was measured using the Geriatric Depression Scale, and life satisfaction, social support and resiliency were measured using validated standard tools. The prevalence of depression was 31.8%. In the adjusted model, factors associated with lower odds of depression were better self-reported health, satisfaction with life (OR=0.08, 95%CI: 0.01–0.46) and high social support (OR=0.23, 95%CI: 0.07–0.78). Resiliency was inversely associated with the odds of depression (low resiliency: OR=4.98, 95%CI: 2.02–12.28; high resiliency: OR=0.21, 95%CI: 0.07–0.64). Given the lack of a basic health profile of this population, this exploratory study is a necessary stepping stone and the first of its kind. This study will help to inform the relevant stakeholders and community leaders and provide baseline evidence for further research and programmatic actions.

